# Potential of *Trichoderma asperellum* as a Growth Promoter in Hydroponic Lettuce Cultivated in a Floating-Root System

**DOI:** 10.3390/plants14030382

**Published:** 2025-01-26

**Authors:** Aldo Gutiérrez-Chávez, Loreto Robles-Hernández, Brenda I. Guerrero, Ana Cecilia González-Franco, Gabriela Medina-Pérez, Angélica Anahí Acevedo-Barrera, Jared Hernández-Huerta

**Affiliations:** 1 Department of Agrotechnological Sciences, Autonomous University of Chihuahua, Campus 1, Av. Pascual Orozco S/N, Chihuahua 31350, Mexico; agutierrezc@uach.mx (A.G.-C.); lrobles@uach.mx (L.R.-H.); brguerrero@uach.mx (B.I.G.); conzalez@uach.mx (A.C.G.-F.); aacevedo@uach.mx (A.A.A.-B.); 2 Institute of Agricultural Sciences, Autonomous University of the State of Hidalgo, Tulancingo de Bravo 43000, Mexico; gabriela_medina@uaeh.edu.mx

**Keywords:** hydroponic system, hydroponic lettuce, plant growth-promoting fungi

## Abstract

The genus *Trichoderma* is widely used in agriculture as a biological agent and biofertilizer, enhancing crop yield and quality. However, its use in hydroponic systems is limited. This study evaluated the potential of *Trichoderma asperellum* as a growth promoter for lettuce (*Lactuca sativa* L.) cv. Starfighter RZ in a floating-root hydroponic system (FHS). *T. asperellum* strains (TaMFP1 and TaMFP2) were isolated from soil and identified morphologically and molecularly. The experiment used a completely randomized design with the following four treatments (*n* = 4): root spraying with TaMFP1, TaMFP2, *T. harzianum* (Trichospore^®^), and uninoculated plants (control). After 30 days, morphological, biochemical, and quality parameters were analyzed. All *Trichoderma* treatments significantly increased plant height (19.0%), root length (25.7%), total fresh biomass (76.4%), total dry biomass (82.63%), and number of leaves (18.18%). The nitrate levels in leaves were unaffected by TaMFP1 and TaMFP2, while Trichospore^®^ reduced the nitrate content by 24.94%. The foliar nitrogen content increased with specific treatments, though the phosphorus and magnesium levels decreased. Visual quality traits, including appearance and firmness, remained unchanged. *T. asperellum* strains TaMFP1 and TaMFP2 enhanced vegetative growth without compromising quality, demonstrating their potential as sustainable tools for hydroponic lettuce production in controlled environments.

## 1. Introduction

The use of biological agents in agriculture has undergone significant growth in recent decades, driven by the need to develop more sustainable production systems that address global challenges, such as soil degradation, climate change, and the increasing demand for food [1,2,3,4,5]. Among these biological agents, fungi of the *Trichoderma* genus have stood out for their versatility and effectiveness, positioning themselves as a key tool in the transition toward more eco-friendly agricultural practices [6,7,8]. *Trichoderma* not only acts as a biofertilizer, enhancing nutrient availability and uptake, but also plays a fundamental role in the biological control of phytopathogens, increasing crop resistance to diseases and environmental stress [9,10,11,12,13]. This biotechnological approach has been widely adopted in both conventional and organic agricultural systems because of its benefits in terms of sustainability, reduced chemical inputs, and improved crop yield and quality [2,11,13]. However, despite its proven advantages in agricultural soils, the potential of *Trichoderma* in hydroponic systems has been little explored, limiting its integration into one of the most promising productive models for the future of agriculture.

Hydroponics has emerged as an innovative and efficient alternative to conventional agricultural systems [14,15]. This approach enables the intensive production of food in limited spaces with significantly lower use of water and chemical inputs, making it an attractive option to address the growing food demand and challenges associated with water scarcity and arable land availability [16,17]. In this context, hydroponics offers a controlled environment that promotes uniform crop growth and minimizes the risks associated with soil-borne diseases [18]. However, despite these advantages, hydroponic systems also present unique challenges, such as the absence of soil microbiota that traditionally contribute to plant health and development [19,20]; this presents an opportunity to study the integration of beneficial microorganisms into these systems to maximize their productivity and sustainability.

The integration of plant growth-promoting fungi into hydroponics can enhance plant development and resistance to biotic factors such as phytopathogens. Additionally, the unique properties of hydroponic systems, such as optimal levels of oxygen and dissolved nutrients in the water, may influence the interaction between plants and beneficial microorganisms [21]. Although the use of *Trichoderma* has been studied in soil systems, with positive results in terms of biomass increase, root development, and stress tolerance [22,23]. There is limited understanding of how these effects can be translated into a hydroponic environment [21,24]. Therefore, the need for more scientific evidence in this field represents a key opportunity to explore new applications of this fungus in a context where the demand for more sustainable and efficient agricultural technologies is becoming increasingly pressing. For this reason, this study aimed to evaluate the potential of *Trichoderma asperellum* as a growth promoter in hydroponically grown lettuce cultivated in a floating-root system.

## 2. Results

### 2.1. Morphological Identification of Trichoderma

**Figure 1:**
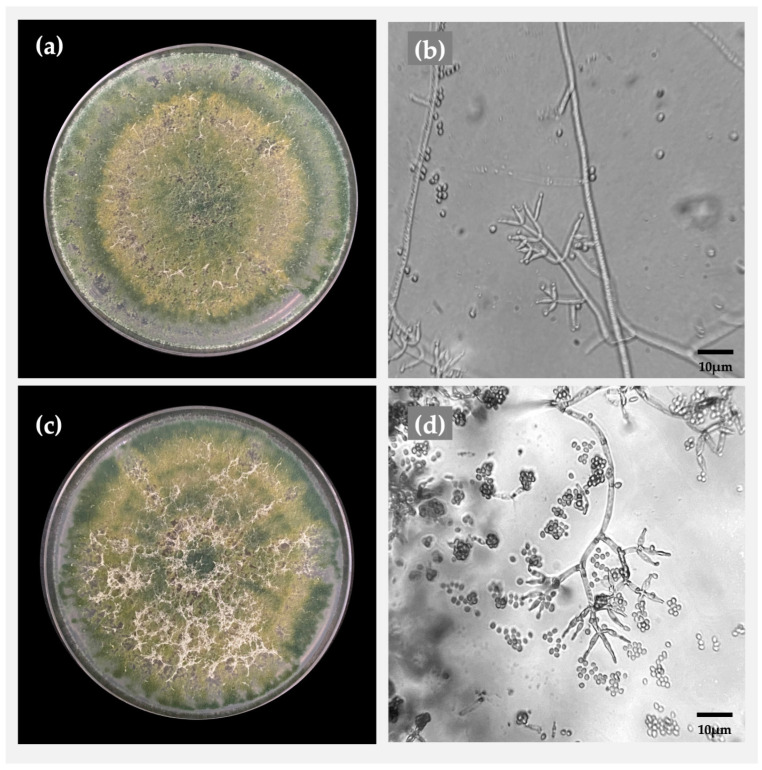
Macro- and microscopic morphologies of *Trichoderma asperellum* strains in potato dextrose agar incubated at 28 °C for 96 h. *T. asperellum* TaMPF1: (**a**) colony; (**b**) presence of phialides and conidia. *T. asperellum* TaMPF2: (**c**) colony; (**d**) presence of phialides and conidia.

The *Trichoderma* strains isolated from the soil showed macroscopic mycelial growth, which expanded rapidly through the medium (Figure 1). The TaMFP1 strain was characterized by a uniform color ranging from yellowish-green to olive-green, with a dense and compact texture and a radial, homogeneous growth pattern. It did not show an abundance of aerial hyphae, giving it a uniform appearance. The edges of the colony were well-defined, with no diffuse areas (Figure 1a). In contrast, the TaMFP2 strain also exhibited a color ranging from yellowish-green to olive-green but with variable intensity. It shows lighter areas and a less compact texture, with branching that gives it a more open appearance. Its growth was also radial but dispersed more toward the edges (Figure 1c). This strain displayed visible aerial hyphae, giving it a cottony appearance. Its edges were less defined and irregular, with zones of texture variation resembling rings, suggesting variable sporulation and less uniform growth than the TaMFP1 strain.

The microscopic characteristics of the isolates corresponded to branched septate hyphae, with the presence of branched hyaline conidiophores (Figure 1b,d). Bottle-shaped phialides were observed at the lateral ends of the conidiophores. The conidia were unicellular and arranged in terminal clusters.

### 2.2. Molecular Identification of Trichoderma

The consensus sequences of the studied internal transcribed spacer (ITS) region were derived from two isolates of *Trichoderma*, identified as TaMFP1 and TaMFP2. The sequence for TaMFP1 had a size of 407 bp (GenBank: PQ786128), while the sequence for TaMFP2 measured 480 bp (GenBank: PQ786130).

Fifty-six sequences from GenBank were obtained through a local alignment, with identity percentages ranging between 98% and 100%. Molecular and bioinformatics analyses using Bayesian methods were conducted, and the average standard deviation of the split frequencies at the end of the 10 million MCMC generations was calculated, with a value of 0.005348. The resulting phylogenetic tree used accession KY687955, corresponding to *T. brevicrassum*; from this point, both isolates, TaMFP1 and TaMFP2, were determined to have 100% identity with *T. asperellum*. GenBank accessions MT529422.1, MT529837.1, and M529846.1, originating from India, confirm in this study that the TaMFP1 and TaMFP2 isolates correspond to this species (Figure S1).

### 2.3. Trichoderma as Lettuce Plant-Growth-Promoting Fungi

**Figure 2:**
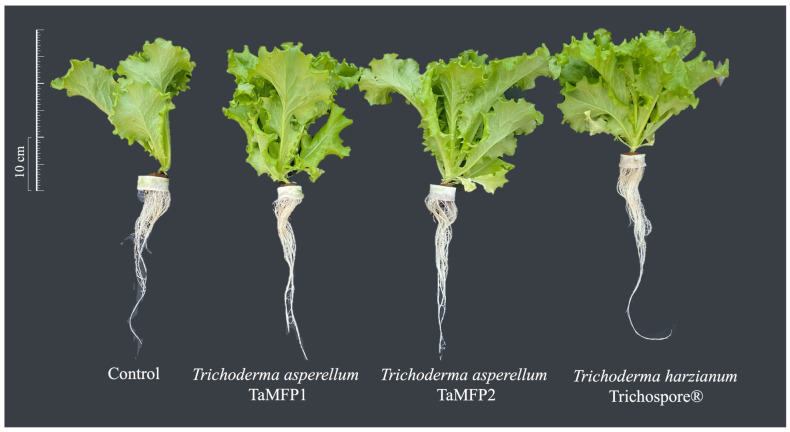
Italian lettuce cv. Starfighter RZ treated with *Trichoderma* spp. cultivated in a floating-root hydroponic system in a greenhouse 30 days post-inoculation. Control = non-inoculated plants.

The results of this study demonstrate that lettuce development in the FHS system was significantly improved by root inoculation with *Trichoderma*. The morphological development of the lettuces treated with the fungi was similar to that of the control plants but with significant differences in the vegetative parameters (*p* < 0.05) (Figure 2 and Table 1).

The fungal treatments improved the plant height by 19.01% compared to the control. Stem length increased by 15.68% with TaMFP1 and Trichospore compared to the control. Root length increased by 25.69% with TaMFP1, TaMFP2, and Trichospore treatments. Trichospore and TaMFP1 increased the leaf area by 33.60% compared to the control. Regarding the leaf number, the fungal treatments significantly increased this parameter by 18.18% compared to the control.

In the case of total fresh biomass, the highest increase occurred with the use of TaMFP1, TaMFP2, and Trichospore, with an improvement of 76.40% compared to the control (Table 2). Likewise, leaf and stem fresh weights improved with fungal treatments by 80.52% and 48.67%, respectively. On the other hand, Trichospore improved the root fresh weight by 75.38% compared to the control.

The highest total dry biomass was achieved using TaMFP1, TaMFP2, and Trichospore, resulting in an 82.63% increase compared to the control. Similarly, the leaf dry weight improved by up to 86.53% with the fungal treatments. The stem and root dry weights improved only by 85.71% and 66.66%, respectively, with the use of Trichospore.

The photosynthetic pigments content was unaffected by the fungal treatments, with values of 1.63 mg g^−1^ FW of chlorophyll a, 0.67 mg g^−1^ FW of chlorophyll b, and 1.57 mg g^−1^ FW of carotenoids (Table 3).

**Figure 3:**
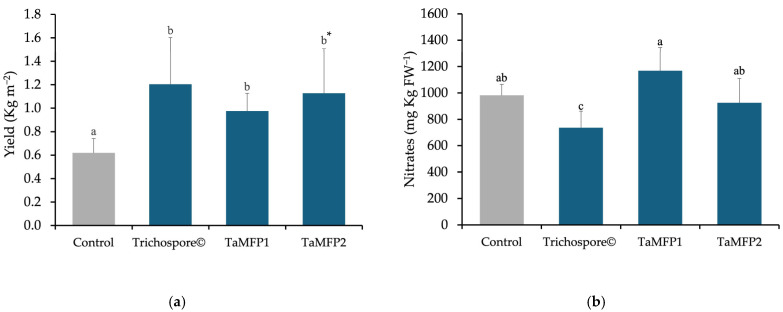
Effect of *Trichoderma* spp. on the yield (**a**) and nitrates content (**b**) of Italian lettuce cv. Starfighter RZ in a floating-root hydroponic system under greenhouse conditions. Control = non-inoculated plants; Trichospore^®^ = commercial product based on *Trichoderma harzianum*; TaMFP1 and TaMFP2 = *T. asperellum*. Bars with the same letter are not statistically different according to the Tukey test or the Games–Howell test *.

The highest leaf yield was achieved using TaMFP1, TaMFP2, and Trichospore, resulting in a 78.49% increase compared to non-inoculated plants, with an average yield of 1.10 kg m^2^ (Figure 3a).

Regarding the nitrate content, the TaMFP1 and TaMFP2 strains did not affect nitrate levels, showing similar values to the control, with an average of 1025.66 mg Kg FW^−1^. However, the Trichospore treatment reduced the nitrate content by 24.94% (Figure 3b).

### 2.4. Analysis Nutrimental

Significant differences (*p* < 0.05) were observed in the levels of macro- and micronutrients present in lettuce shoots, but these differences were only evident in certain specific elements (Table 4).

Using *Trichoderma* treatments did not increase the N content compared to the control. However, differences were observed among the treatments. Trichospore and TaMFP2 exhibited 20.94% more N content than TaMFP1. The P content was statistically similar between the control and the Trichospore treatment; however, TaMFP1 and TaMFP2 exhibited significant decreases, with reductions of 28.74% compared to the control. No significant differences were observed in the K and Ca contents among the treatments, with averages of 4.53% and 1.36%, respectively. The control presented the highest Mg level, while all *Trichoderma* treatments showed reductions of up to 20.70% compared to the control.

Regarding micronutrients, the control treatment showed the highest Fe content, while the fungal treatments decreased this element by up to 38.02%. The Mn content was reduced by Trichospore and TaMFP1 by 28.61%, compared to the control. Finally, the Cu and Zn levels showed no significant differences among treatments, with an average of 10.84 ppm and 59.49 ppm, respectively.

All treatments showed variations in macronutrient levels compared to the ideal ranges for the optimal nutrition in hydroponic lettuce culture. The N-total and K levels in all treatments did not reach the sufficiency ranges. In contrast, the P levels in all treatments were above the sufficiency ranges. Only the Ca and Mg levels were within the sufficiency ranges. Concerning micronutrient content, most of the treatments exhibited nutrient levels that were within the optimal range. Only the control group showed an Fe level above the established recommendations.

### 2.5. Lettuce Quality

**Figure 4:**
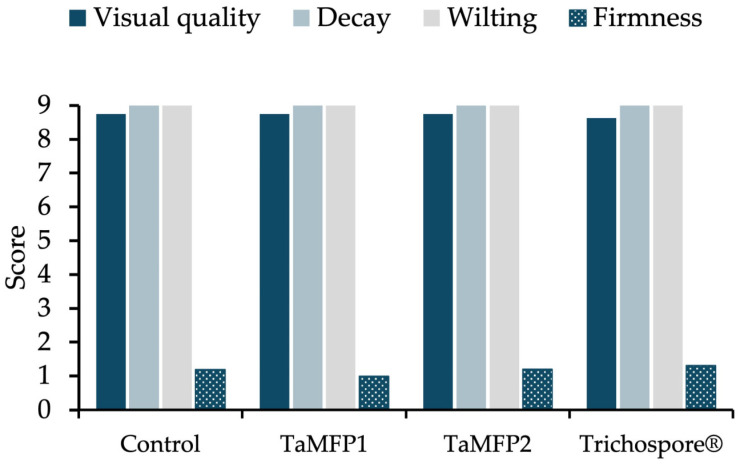
Effect of *Trichoderma* spp. on the quality parameters of Italian lettuce cv. Starfighter RZ in a floating-root hydroponic system under greenhouse conditions. Control = non-inoculated plants; Trichospore^®^ = commercial product based on *Trichoderma harzianum*; TaMFP1 and TaMFP2 = *T. asperellum*.

The quality of the lettuce produced in this trial, under both fungal treatments and the non-inoculated plants, showed no statistically significant differences (Figure 4). The average score for visual quality was 8.7, indicating excellent-looking lettuce, essentially free from defects. For the parameters of decay and wilting, all treatments achieved the maximum score (9), indicating the absence of these issues. Finally, the firmness of the lettuce had an average score of 1.2, characterizing the lettuce as soft, easily compressed, or spongy, a characteristic inherent to the variety used in this study.

### 2.6. Principal Component Analysis

**Figure 5:**
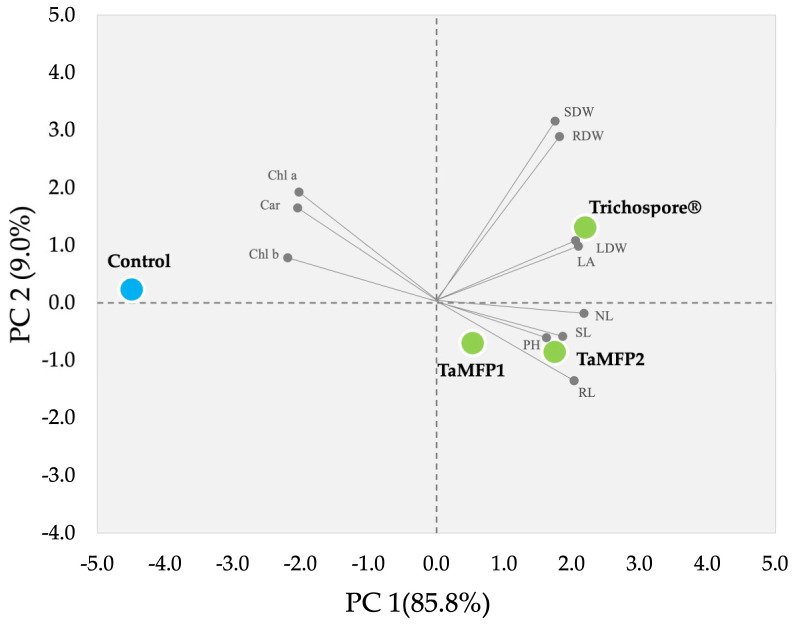
Principal component analysis of the growth promotion in Italian lettuce plants cv. Starfighter RZ, treated with *Trichoderma* under a greenhouse floating-root hydroponic system (KMO 0.80, X^2^ = 760, *p* < 0.001). PC 1 and PC2 = principal components; NL = number of leaves; LA = leaf area; SL = stem length; RL = root length; RDW = root dry weight; SDW = stem dry weight; LDW = leaf dry weight; PH = plant height; Chl b = chlorophyll b; Chl a = chlorophyll a; Car = carotenoids.

In the principal component analysis conducted to evaluate the effect of *Trichoderma* treatments on the growth of lettuce cultivated in a floating-root hydroponic system under greenhouse conditions, significant variation patterns were observed among treatments (Figure 5). The first two components (PCs) explained 94.8% of the variance, with PC 1 at 85.6% and PC 2 at 9.0%. The *Trichoderma* treatments clustered in the right quadrant, as shown in Figure 5, indicating a clear differentiation compared to the control group, which was positioned at the far left of the PC 1 axis.

The Trichospore treatment was positively associated with growth variables such as RDW, SDW, LDW, and LA, while the TaMFP1 and TaMFP2 treatments were more closely related to PH, SL, NL, and RL. On the other hand, the control group was characterized by being distanced from most growth variables, suggesting a lesser effect on vegetative development than the *Trichoderma* treatments.

## 3. Discussion

*Trichoderma* is a genus of ubiquitous filamentous fungi that is found in soil, decaying plant matter, and the rhizospheres of plants [26]. *Trichoderma* can benefit plants when associated with them by acting as a biocontrol agent against phytopathogens or by stimulating plant growth [27]. In this study, the macroscopic and microscopic characteristics of *Trichoderma* align with those reported by Sánchez-García et al. [28] and Zapata-Sarmiento et al. [29], who observed mycelial growths of *T. asperellum* on PDA media with yellow–green and green coloration, which is characteristic of this species [26]. Moreover, ITS sequencing has proven effective for identifying *Trichoderma* species. Studies have successfully utilized ITS1 region amplification and sequencing to identify *T. harzianum* and *T. erinaceum* isolates from North Bengal [30]. Based on the results of this study, it is evident that root inoculation of lettuce plants with *Trichoderma* in a floating hydroponic system significantly improved their development and various vegetative parameters. These results are similar to those reported by Leu et al. [31], who observed a significant increase in plant height (95.4%) and leaf size (35.7%) when inoculating basil (*Ocimun basilicum* L.) grown in FHS with *T. atroviride*. Similarly, Moreira et al. [24] reported a 20% increase in root length when lettuce grown in nutrient film technique (NFT) systems was inoculated with *T. harzianum* via foliar application. Yedidia et al. [32] observed a 3.2-fold increase in the number of leaves in cucumber (*Cucumis sativum* L.) plants inoculated with *Trichoderma* sp. Likewise, Pineda-Acosta et al. [33] reported an 11% increase in the number of leaves in lettuce cultivated in FHS when *T. harzianum* was applied. Regarding biomass, Oliveira et al. [34] reported significant increases in shoot fresh weight (24.4%) and root fresh and dry weights (74.5% and 544%, respectively) when arugula (*Eruca vesicaria* L.) grown in NFT systems was inoculated with *T. harzianum*. Pineda-Acosta et al. [33] consistently reported a 63% increase in lettuce foliage biomass in FHS when *T. harzianum* was employed.

The increase in lettuce growth due to *Trichoderma* may result in its ability to stimulate the development of primary meristematic tissues and increase the volume and number of root hairs, thereby improving water and nutrient absorption [35,36]. This mechanism supports the robust growth observed in lettuce seedlings treated in this study. Additionally, Garnica-Vergara et al. [37] noted that *Trichoderma* influences root architecture by stimulating auxin signaling, enhancing the growth of primary and secondary roots. This alteration in root structure contributes to greater nutrient uptake efficiency, which aligns with the root development enhancements observed in this study. Moreover, Vargas et al. [38] reported that colonization of the rhizosphere by *T. virens* in maize plants increases photosynthesis rates and improves CO_2_ uptake in leaves, promoting plant growth. Although photosynthesis rates were not directly measured in this study, the increased leaf area and number of leaves could be interpreted as indirect evidence of enhanced photosynthetic activity facilitated by *Trichoderma* treatment. The biomass increase observed might be attributed to improved nutrient absorption and utilization efficiency, resulting in vigorous growth. This effect is supported by *Trichoderma’s* ability to produce secondary metabolites and enzymes that promote root health and enhance overall nutrient use efficiency, as suggested by Goswami et al. [39] and Jalal et al. [40].

The improvement in nutrient uptake, such as phosphorus (P), sulfur (S), zinc (Zn), and iron (Fe), could partially explain the biomass increases observed in this study. *Trichoderma* produces secondary metabolites that can act as carriers for nutrients like iron and zinc [39,40]. This facilitates nutrient absorption by roots and their subsequent translocation to shoots. In this study, *Trichoderma* significantly affected certain nutrient levels in lettuce tissues, including nitrogen and some micronutrients. Garnica-Vergara et al. [37] highlighted *Trichoderma’s* capacity to modify root architecture and improve hormonal signaling, enhancing essential nutrient uptake. Additionally, *Trichoderma’s* ability to produce phytohormones such as indole-3-acetic acid (IAA) and other growth-promoting secondary metabolites [41,42] contributes to the improved nutrient acquisition and efficiency observed in treated plants.

In terms of nitrate content, the lettuce in this study exhibited levels below the limits established by European Union Regulation 1258/2011, which sets a maximum NO_3_ limit for summer-grown greenhouse lettuce at 2500–3500mg/kg fresh weight. Notably, *Trichoderma harzianum*’s (commercial strain) application reduced nitrate levels compared to the control. This finding aligns with Oliveira et al. [34], who reported up to a 70.17% reduction in nitrate content in hydroponic arugula inoculated with *T. harzianum*. However, recent studies, such as Patlokavá and Pokluda [43], found that *T. harzianum* inoculation in an aquaponic system increased the nitrate content in basil by 36.4%.

These discrepancies in *Trichoderma’s* effects may result from variability in specific interactions between the fungus and plant species under different cultivation conditions [10]. The nitrate reduction observed in this study could be related to the ability of certain *Trichoderma* species, like *T. asperellum*, to enhance nitrogen-use efficiency in crops. Studies have shown that *Trichoderma* can regulate the expression of genes associated with nitrate transporters (NRT2.1 and NRT2.2) and nitrate reductase activity, potentially explaining reduced nitrate accumulation in foliar tissues [44]. This outcome is significant as lower nitrate accumulation benefits the nutritional quality and consumer health.

Regarding crop yield, the results of this study surpassed those reported for field conditions where lettuce varieties inoculated with *Trichoderma* spp. demonstrated yield increases. For instance, *T. harzianum* ESAQ1306 was particularly effective, boosting productivity by up to 66% compared to controls [45]. *T. asperelloides* also showed increases, with tiled improvements of up to 22% in various lettuce varieties [46].

The visual quality of the lettuce produced in this trial showed no statistically significant differences, which is a positive outcome, since visual quality is crucial for successful commercialization. Factors influencing visual quality include nutrient management, and the optimal nitrogen and phosphorus levels in this study were slightly below the recommended range, and no deficiencies were observed. Phosphorus levels exceeded recommended values across all treatments. Moreover, no significant differences were found in photosynthetic pigments, which is notable as chlorophyll content in acuter leaves influences visual quality [33,47]. This also implies reduced synthetic fertilizer requirements, contributing to more sustainable and environmentally responsible agricultural practices [39].

Future research should explore how *Trichoderma* directly affects photosynthesis, hormonal profiles, and primary metabolism in hydroponic crops and its interactions with other microorganisms in the root microbiome. It would also be valuable to assess its impact on the nutritional and functional qualities of crops, including bioactive compounds, and to analyze the variability of its effects across different species, strains, and cultivation systems.

## 4. Materials and Methods

Unless otherwise specified, reagents and biochemical tests were purchased from Sigma-Aldrich Química de México (Toluca de Lerdo, Edo Mex, Mexico). Molecular biology assay reagents were obtained from Promega Corporation (Madison, WI, USA).

### 4.1. Localization

The trial was conducted in the Applied Microbiology, Plant Pathology, and Postharvest Physiology Laboratory (MAFFP) and the greenhouse of the Faculty of Agrotechnological Sciences (FACIATEC), Autonomous University of Chihuahua (UACH), Chihuahua, MX (28°39’24” N 106°05’12” W). It lasted from 2 February to 3 April 2023, with 21 days in the seedling production stage and 30 days from the beginning of hydroponic culture until harvest.

### 4.2. Samples Collection

Soil samples were collected from Scholar Orchard FACIATEC, UACH in Chihuahua, Chihuahua, Mexico (28°39′27.2″ N 106°05′15.1″ W). Sampling was performed according to Ha [48], selecting five diagonal points, and 500 g of soil was collected at a depth of 20 cm at each point. Then, samples were placed in new plastic bags, labeled, and transported to the MAFFP, UACH, where they were kept at 4 °C until use.

### 4.3. Trichoderma Strain Isolation

Isolation was carried out using serial dilutions from 10^−1^ to 10^−4^. Ten grams of soil was placed in sterile tubes with 90 mL of sterile distilled water and 1% Tween^TM^ 20 and shaken for 5 min in a vortex at a speed of 4 (Daigger Vortex-Genie 2; Scientific Industries Inc., Bohemia, NY, USA). A total of 100 μL was taken from each tube and distributed in a Petri dish with Potato Dextrose Agar (PDA; BD Difco Laboratories, Sparks, Maryland, MD, USA) with 0.05% lactic acid (Faga Lab, Ciudad de Mexico, Mexico) and placed in incubation at 28 °C for seven days or until the appearance of *Trichoderma* spp. Purification was carried out by the hyphal tip technique [49], transferring the fungus to the PDA medium.

### 4.4. Morphological Identification

The strains were identified by observing the macroscopic characteristics in PDA and via microscopic observations by staining the fungal colonies with lactophenol cotton blue. The characteristics were then compared with identification keys from the American Society of Phytopathology [50].

### 4.5. Extraction, Amplification, and Sequencing of DNA

DNA was obtained using the cetyltrimethylammonium bromide (CTAB) method [51]. The DNA was quantified using a Nanodrop 2000c (Thermo Scientific, Waltham, MA, USA). Dilutions of each sample were prepared to 20 ng. The ITS was amplified using ITS5/ITS4 primers [52]. For amplification of the region, the PCR reaction mix was prepared to a final volume of 13 μL containing 1× Taq polymerase enzyme buffer, 0.8 mM deoxy nucleoside triphosphates (0.2 mM each), 100 ng DNA, 20 pmol of each primer, and 2 units of GoTaq DNA polymerase (Promega, Madison, WI, USA).

Amplifications were performed with an initial denaturation cycle at 95 °C for 4 min, followed by 35 cycles of denaturation at 95 °C for 1 min, primer annealing at 57 °C for 1 min, and a final extension at 72 °C for 2 min. The PCR reactions were carried out in a Peltier Thermal Cycler PTC-200 (Bio-Rad, Ciudad de Mexico, Mexico), and the amplifications were verified by electrophoresis on a 1.5% agarose gel that was prepared with 1× TAE buffer (Tris Acetate-EDTA) and run at 95 V cm^−3^ for 1 h. The gel was stained with Gel Red (Biotium, Fremont, CA, USA), and the bands were visualized on a transilluminator (Infinity 3000 Vilber, Lourmat, Eberhardzell, Germany). Amplified products were purified with the ExoSAP kit (Affymetrix, Santa Clara, CA, USA), following the manufacturer’s instructions. These products were sequenced on an Applied Biosystems 3730XL model (Applied BioSystems, Waltham, MA, USA).

### 4.6. Phylogenetic Reconstruction

The sequences of both strands were analyzed, edited, and assembled using the BioEdit version 7.0.5 software [53] to generate a consensus sequence of the ITS region for the species in question. These consensus sequences were compared with those deposited in GenBank at the National Center for Biotechnology Information (NCBI) using the BLASTn 2.2.19 tool [54], considering those with identity percentages above 95%. Multiple alignment was performed using MEGA v10 software [55], based on the taxonomic sampling by Gu et al. [56], integrating the ITS sequences of the species in question.

The Hasegawa–Kishino–Yano Invergamma (HKY + G + I) nucleotide substitution model was identified as the best fit for the data using the JModelTest 2 software [57]. Phylogenetic analysis was carried out using MCMC Bayesian inference with the MrBayes v3.2.6 × 64 program [58] in two independent runs of MC3 chains and 10,000,000 generations (standard deviation ≤ 0.01), with a sampling frequency of every 1000 generations. Additionally, four chains were established with two nucleotide substitution rates and a burn-in of 25%. The convergence of the chains was visualized in Tracer v. 1.7.2 [59]. The generated tree was observed using FigTree v 1.4.4 software [59].

### 4.7. Seedling Production

The lettuce variety (*Lactuca sativa* L.) used in this study was the cultivar Starfighter RZ (81–85) (Rijk Zwaan^®^, De Lier, The Netherlands), a Batavia-type lettuce known for its high adaptability. It is resistant to *Bremia lactucae* (Bl:3032EU/Bl:7-9US), *Nasonovia ribisnigri* (Nr:0), and *Fusarium oxysporum* (Pb). Additionally, it exhibits tolerance to internal tip burn and bolting, making it an excellent choice for commercial cultivation (www.rijkzwaan.com (accessed on 29 December 2024)).

Pelleted seeds of lettuce were sown in low-density polyurethane foam squares (5 cm^2^ × 2.5 cm thick). Subsequently, they were placed in a plant growth room with a photoperiod of 16 h of light at 28 °C and 8 h of darkness at 18 °C and 80 ± 2% relative humidity (RH). Seeds were irrigated every four days using purified water, and following germination, they were nourished with Steiner nutrient solution (Inverfarms^®^, Querétaro, Mexico), which was composed of (ppm): 126 of NO_3_, 42 of NH_4_, 31 of PO_4_, 274 of K^+^, 181 of Ca^2+^, 48.6 of Mg^2+^, 112 of SO_4_, 1.3 of Fe-EDTA, 0.8 of Mn-EDTA, 0.3 of Zn-EDTA, 0.06 of Cu-EDTA, 0.4 of B, and 0.06 of Mo. The pH was maintained at 6.0–6.5, with electrical conductivity (EC) at 1.5–2.5 mS/cm [60].

### 4.8. Preparation of Inoculum

*Trichoderma* strains, including isolates and *T. harzianum* (Trichospore), were cultured on PDA in Petri dishes at 28 °C for seven days. After incubation, fungal conidia were collected by scraping the mycelial surface with a sterile spatula. The collected conidia were then suspended in 20 mL of sterile water and filtered through a syringe equipped with glass fiber filters to isolate the conidia. Conidial concentration was determined using a Neubauer counting chamber (Weber Scientific International Ltd., Teddington, UK) and adjusted to 1 × 10^6^ conidia/mL with 0.03% (*w*/*v*) xanthan gum [61].

### 4.9. Trial Establishment

The 21 d lettuce seedlings were root inoculated with 6 mL of the conidial suspensions (1 × 10^6^ conidia/mL) by spraying (atomizer-JR-24/410, MultiPlastic^®^, Tlajomulco de Zuñiga, Mexico) three times at 8-day intervals. The inoculated seedlings were placed in individual FHS, which consisted of rectangular heavy-duty polypropylene containers of 19.0 gallon (72 L) (17.3 in. W × 24.8 in L × 14.9 in H, Atlanta Model S019TZZ0, PlasticTrends^®^, Guadalajara, Tlajomulco de Zuñiga, Mexico). The system-cultivate area consisted of one-inch-thick polystyrene plates sized 15.7 in. W × 23.6 in. L, which were placed inside each containment. Each plate had five holes of 5 cm in diameter, equidistantly spaced at 20 cm, for seedling placement.

The oxygenation of the FHS was supplied through a horizontal air compressor 25L, 3 HP, 127V (15006/COMP-25LT, Truper^®^, Jilotepec, Mexico), connected to a manual timer (15 min every 6 h throughout the trial) (B012890E7Y, Volteck^®^, Ciudad de Mexico, Mexico). For this purpose, an oxygenation system consisting of three adjustable drippers 0–70 L/h (B07BTDDDJKJ, Zerodis, Hydroenviroment^®^, Tlanepantla, Mexico) equidistantly distributed and interconnected with micro tubing of 4/7 mm (Hydroenviroment^®^, Tlanepantla, Mexico) to the air compressor was placed at the bottom of each container. The Steiner nutrient solution was used as a nutrient source with the pH maintained at 6.0–6.5 and EC at 1.5–2.5 mS/cm [60]. The trial was stabilized under greenhouse conditions (25.4 °C and 27.7 to 71.2% RH).

### 4.10. Plant Analysis

#### 4.10.1. Morphological Variables

Plant morphological variables, such as plant height, stem, root length, number of leaves per head, leaf area (Canopeo^®^ phone app [62]), and fresh and dry biomass, were evaluated after 30 d post inoculation with the fungus. The fresh and dry biomass of leaves, stem, root, and total plants were evaluated separately. The dry biomass was determined after being dried in a forced-air convection oven (SMO3, Shel Lab^®^, Cornelius, OR, USA) at 75 °C to a constant weight. The biomass was measured with an analytical balance (XT-220A, Precisa Instruments ^®^, Zurich, Switzerland). The weights are expressed as grams per plant (g plant^−1^).

#### 4.10.2. Photosynthetic Pigments

Quantification of the photosynthetic pigments, such as chlorophyll a, chlorophyll b, and carotenoids, were determined using the Lichtenthaler and Wellburn [63] methodology, after 30 d post-inoculation with the fungus. The pigments were extracted from fresh leaves (0.1 g) by homogenizing with 4 mL of 80% acetone (*v*/*v*, Sigma-Aldrich, St. Louis, MI, USA) followed by centrifuging at 3000 rpm for 5 min. The absorbance supernatant was measured with a UV-visible spectrophotometer (Evolution 60S, Thermo Scientific^®^, Madison, WI, USA) at 663, 645, and 470. The concentrations of photosynthetic pigments were calculated with the following formulas:

(1) $$\mathrm{Chlorophy}ll\;a\;(\mathrm{mg}g^{-1}\mathrm{FW})=\left(12.21\;\times \;A_{663}\;-2.81\;\times \;A_{645}\right)\;\times \;V/(1000\;\times \;W)$$

(2) $$\mathrm{Chlorophyl}l\;b\left(\mathrm{mg}g^{-1}\mathrm{FW}\right)=\left(20.13\;\times \;A_{645}\;-5.03\;\times \;A_{663}\right)\;\times \;V/(1000\;\times \;W)$$

(3) $$\mathrm{Carotenoids}\;\left(\mathrm{mg}\;g^{-1}\mathrm{FW}\right)=\left\lfloor \frac{\left(1000\;\times A_{470}-3.27\;\times {\mathrm{Chl}}_{a}-104\;\times {\mathrm{Chl}}_{b}\right)}{229}\right\rfloor \times \;V/(1000\;\times \;W)$$

where V = volume of 80% (*v*/*v*) acetone (mL), and W = fresh weight (FW) of the samples (g).

#### 4.10.3. Foliar Nutrients

Leaf tissue from each treatment was oven-dried at 68 °C for 48 h, then ground in a Wiley mill (Thomas Scientific 800-345-2100, Swedesboro, NY, USA) to a 1 mm mesh size to determine nutrients after 30 d post inoculation with the fungus. Four replicates were taken from each treatment for nutrient analysis.

The N-total (%) was quantified using the Kjeldahl method which uses a Novatech^®^ digester and Labconco^®^ Micro Kjeldahl rapid distillation unit [64].

The contents of Cu^2+^, Fe^2+^, Mn^2+^, and Zn^2+^ were determined by 25 mL triacid digestion (HNO_3_, HClO_4_, and H_2_SO_4_ in a 10:1:0.25 ratio) using a 0.1 g dry sample [65]. Concentrations were determined using an atomic absorption spectrophotometer (Perkin Elmer Analyst 100, Waltham, MA, USA) and expressed in mg Kg^−1^.

The contents of Ca^2+^, Mg^2+^, and K^+^ were determined using samples from the previous digestion diluted to 1% in deionized H_2_O. Quantifications were performed using an atomic absorption spectrophotometer (Perkin Elmer Analyst 100, Waltham, MA, USA) and expressed in percentage.

The P-total (%) content was determined by the vanadate–molybdate method. The determination was carried out by UV-visible spectrophotometry (Evolution 60S, Thermo Scientific^®^, Madison, WI, USA) at 410 nm after 30 min of color development [66].

#### 4.10.4. Nitrate Content

The determination of nitrates was carried out in dry matter of leaves by extraction with water and based on the nitration of salicylic acid, according to the methodology proposed by Cataldo et al. [67], by boiling 0.5 g of the sample for 30 min in 5 mL of distilled water to obtain the extract. After cooling, the extract was filtered with filter paper (Whatman ^TM^ No1, Whatman North America, Florham Park, NJ, USA). Then, 0.8 mL of 5% (*w*/*v*) salicylic acid in concentrated H_2_SO_4_ was added to 0.2 mL of the extract. After 20 min, 19 mL of 2N NaOH solution was added. The samples were measured at 410 nm with UV-visible spectrophotometry, and the nitrate concentration was calculated from a KNO_3_ standard curve (10, 20, 30, 40, 50, 75, and 100 μg/g).

#### 4.10.5. Crop Yield

The yields of the lettuce was calculated according to the formula proposed by Moreira et al. [24], as follows:

(4) $$Yield\left(kgm^{2}\right)=Shoot\;fresh\;weight\;\left(kg\right)\;\times \;plant\;population\;per\;m^{2}\;(25\;plants\;m^{2})$$

#### 4.10.6. Quality Parameters

The quality of the lettuce was evaluated qualitatively using the scale proposed by Kader et al. [68] with some modifications. Firmness, visual appearance, decay, and wilting were assessed (Table 5).

### 4.11. Statistical Analysis

The trial was established under a completely randomized experimental design with four treatments and four replications, where each repetition consisted of four plants. Treatments consisted of plants inoculated with *T. asperellum* TaMFP1, seedlings inoculated with *Trichoderma* TaMFP2, seedlings inoculated with *T. harzianum* of the commercial product Trichospore^®^ (Grow Depot Mexico, Torreon, Mexico), and as a control non-inoculated plants (control). The two isolates selected for this study were previously evaluated in experiments conducted in the MAFFP. These tests consistently demonstrated their ability to stimulate plant growth and act as biocontrol agents in economically important crops such as pepper (*Capsicum annuum* L.) and tomatoes (*Solanum lycopersicum* L.). Although the results of these studies have not yet been published, they served as the basis for selecting these isolates for the present research.

The data from *Trichoderma*-induced lettuce plant growth in the FHS were subjected to Shapiro–Wilk tests to determine their normal distribution and the Levene test to determine the homogeneity of variance. Then, the data were analyzed by analyses of variance (ANOVA)/Tukey test, Welch’s ANOVA/Games–Howell test, or Kruskal–Wallis/Conover–Iman test (*p* < 0.05) according to the Shapiro–Wilk and Levene tests. A principal component analysis (PCA) plot was conducted to find the differences among treatments. Prior to this, Bartlett’s efficiency test was performed to test the validity of the data set (*p* < 0.01). The suitability of the data set to perform the PCA was tested using the Kaiser–Meyer–Olkin (KMO) test, which considered KMOs greater than 0.60 acceptable [69]. All analyses were performed with the InfoStat software (InfoStat version 2009; InfoStat Group, Cordoba, Argentina) and JAMOVI 2.5.2.0 software.

## 5. Conclusions

This study evaluated the efficacy of *Trichoderma asperellum* strains (TaMFP1 and TaMFP2) as growth promoters in hydroponic lettuce (*Lactuca sativa* cv. Starfighter RZ) cultivated in a floating-root system. The hypothesis posited that these strains could enhance growth parameters without compromising plant quality. The findings confirm this, demonstrating significant improvements in morphological and biomass parameters while maintaining visual and biochemical quality. These results underscore the potential of *T. asperellum* as a sustainable growth promoter in hydroponic lettuce systems, contributing to sustainable agriculture practice by integrating biological agents into controlled environments. The growth-promoting effects may extend beyond nutrient optimization, potentially involving hormonal modulation or enhanced root–microbe interactions. Future research could unveil novel mechanisms of action, broadening the application of *T. asperellum* in sustainable agriculture.

## Figures and Tables

**Table 1:** Effect of *Trichoderma* spp. on the growth of Italian lettuce cv. Starfighter RZ in a floating-root hydroponic system under greenhouse conditions.

Parameter ^1^	Treatments			
	Control	*T. asperellum* TaMFP1	*T. asperellum* TaMFP2	Trichospore^®^
PH (cm)	20.41 ± 2.90 ^b^	23.58 ± 2.35 ^a^	24.95 ± 2.90 ^a^	24.34 ± 2.53 ^a^
SL (cm)	6.19 ± 0.46 ^b^	7.42 ± 1.01 ^a^	6.90 ± 1.38 ^a^	7.17 ± 0.56 ^ab^
RL (cm)	29.39 ± 5.13 ^b^	36.56 ± 4.78 ^a^	37.77 ± 8.67 ^a^	36.48 ± 7.96 ^a^
LA (cm^2^ plant^−1^) *	814.19 ± 133.76 ^b^	932.55 ± 162.81 ^ab^	1076.58 ± 299.21 ^a^	1098.96 ± 309.07 ^a^
NL (plant^−1^) **	13.31 ± 2.33 ^b^	15.50 ± 2.31 ^a^	15.75 ± 2.77 ^a^	15.94 ± 1.29 ^a^

^1^ The results are the mean ± S.D. of four plants per replicate, with four replicates. Different superscript letters in the same row indicate significant differences according to the Tukey test, Games–Howell test *, or Conover–Iman ** test at the 0.05 level. Control = non-inoculated plants; Trichospore^®^ = commercial product based on *Trichoderma harzianum;* PH = plant height; SL = stem length; RL = root length; LA = leaf area; NL = number of leaves.

**Table 2:** Effect of *Trichoderma* spp. on the biomass of Italian lettuce cv. Starfighter RZ in a floating-root hydroponic system under greenhouse conditions.

Treatment ^1^	Control	*T. asperellum* TaMFP1	*T. asperellum* TaMFP2	Trichospore^®^
Fresh biomass (g plant^−1^)				
Leaves	22.99 ± 4.75 ^b^	36.79 ± 5.96 ^a^	42.39 ± 14.29 ^a^	45.33 ± 15.09 ^a^
Stem	1.76 ± 0.43 ^b^	2.23 ± 0.45 ^a^	2.74 ± 1.02 ^a^	2.88 ± 0.94 ^a^
Root *	2.64 ± 0.98 ^b^	4.04 ± 1.44 ^ab^	3.93 ± 1.81 ^ab^	4.63 ± 1.96 ^a^
Total	27.39 ± 5.32 ^b^	43.06 ± 6.98 ^a^	49.06 ± 16.76 ^a^	52.83 ± 17.09 ^a^
Dry biomass (g plant^−1^)				
Leaves	1.04 ± 0.31 ^b^	1.77 ± 0.40 ^a^	1.87 ± 0.65 ^a^	2.18 ± 0.63 ^a^
Stem	0.09 ± 0.03 ^b^	0.10 ± 0.02 ^b^	0.12 ± 0.05 ^ab^	0.15 ± 0.05 ^a^
Root *	0.07 ± 0.03 ^b^	0.10 ± 0.04 ^ab^	0.09 ± 0.04 ^ab^	0.13 ± 0.03 ^a^
Total	1.19 ± 0.34 ^b^	1.98 ± 0.44 ^a^	2.08 ± 0.71 ^a^	2.46 ± 0.68 ^a^

^1^ The results are the mean ± S.E. of four plants per replicate, with four replicates. Different superscript letters in the same row indicate significant differences according to the Tukey test * or the Games–Howell test at the 0.05 level. Control = non-inoculated plants; Trichospore^®^ = commercial product based on *Trichoderma harzianum.*

**Table 3:** Effect of *Trichoderma* spp. on the photosynthetic pigments of Italian lettuce cv. Starfighter RZ in a floating-root hydroponic system under greenhouse conditions.

Treatment ^1^	Control	*T. asperellum* TaMFP1	*T. asperellum* TaMFP2	Trichospore^®^
Photosynthetic pigments (mg/g g FW^−1^)				
Chlorophyll a	1.80 ± 0.18 ^a^	1.67 ± 0.27 ^a^	1.58 ± 0.27 ^a^	1.66 ± 0.29 ^a^
Chlorophyll b	0.74 ± 0.08 ^a^	0.68 ± 0.11 ^a^	0.66 ± 0.10 ^a^	0.67 ± 0.12 ^a^
Carotenoids	1.73 ± 0.15 ^a^	1.58 ± 0.26 ^a^	1.54 ± 0.21 ^a^	1.59 ± 0.28 ^a^

^1^ The results are the mean ± S.E. of four plants per replicate, with four replicates. Different superscript letters in the same row indicate significant differences according to the Tukey test at the 0.05 level. Control = non-inoculated plants; Trichospore^®^ = commercial product based on *Trichoderma harzianum.*

**Table 4:** Effect of *Trichoderma* spp. on the macro- and micronutrients of Italian lettuce cv. Starfighter RZ in a floating-root hydroponic system under greenhouse conditions.

Treatment ^1^	Control	Trichospore^®^	TaMFP1	TaMFP2	Sufficiency Range ^2^
Macronutrients (%)					
N	3.31 ± 0.08 ^ab^	3.70 ± 0.13 ^a^	3.08 ± 0.27 ^b^	3.75 ± 0.29 ^a^	4.5–6.5
P	1.27 ± 0.21 ^a^	1.24 ± 0.10 ^a^	0.91 ± 0.02 ^b^	0.90 ± 0.09 ^b^	0.3–0.8
K	4.43 ± 0.76 ^a^	4.67 ± 0.39 ^a^	4.98 ± 0.36 ^a^	4.05 ± 0.22 ^a^	6–10
Ca *	1.52 ± 0.26 ^a^	1.32 ± 0.06 ^ab^	1.38 ± 0.02 ^a^	1.24 ± 0.08 ^a^	1–2
Mg	0.66 ± 0.01 ^a^	0.52 ± 0.03 ^b^	0.53 ± 0.03 ^b^	0.52 ± 0.04 ^b^	0.35–0.75
Micronutrients (ppm)					
Fe	287.0 ± 32.35 ^a^	167.87 ± 5.20 ^b^	180.87 ± 18.95 ^b^	184.87 ± 11.62 ^b^	50–200
Mn	103.75 ± 10.13 ^a^	67.37 ± 6.90 ^c^	80.75 ± 8.79 ^bc^	90.75 ± 8.45 ^ab^	20–200
Cu	11.50 ± 1.77 ^a^	10.12 ± 2.78 ^a^	10.75 ± 1.70 ^a^	11.00 ± 0.91 ^a^	5–15
Zn	66.25 ± 6.30 ^a^	54.37 ± 2.49 ^a^	60.87 ± 8.01 ^a^	56.50 ± 4.77 ^a^	20–75

^1^ The results are the mean ± S.E. of four replicates. Different superscript letters in the same row indicate significant differences according to the Tukey test or Conover–Iman * test at the 0.05 level. Control = non-inoculated plants; Trichospore^®^ = commercial product based on *Trichoderma harzianum;* TaMFP1 and TaMFP2 = *Trichoderma asperellum*. ^2^ Values corresponding to lettuce grown under greenhouse conditions, according to Campbell [25].

**Table 5:** Rating scale for scoring the visual quality of the harvested lettuce.

Trait	Score	Description
Firmness description	1	Soft, easily compressed, or spongy
	2	Fairly firm, neither soft nor firm, good head formation
	3	Firm, compact but may yield slight to moderate pressure
	4	Hard, compact, and solid
	5	Extra hard, over-mature, may have cracked mid ribs
Visual quality	1	Extremely poor, disposable
	3	Poor, many defects, limit of salability
	5	Fair, slightly to moderately defects, lower limit of sales appeal
	7	Good, minor defects
	9	Excellent, essentially free from defects
Decay	1	Extreme, disposable
	3	Severe, salvageable but usually not salable
	5	Moderate, objectionable, definitely impairs salability
	7	Slight, slightly objectionable, may impair salability
	9	None
Wilting	1	Extreme, not acceptable under normal conditions
	3	Severe, definitely objectionable
	5	Moderate, becoming objectionable
	7	Slight, not objectionable
	9	None, fresh cut appearance

## Data Availability

The data presented in this study are available upon request from the corresponding authors.

## Supplementary Materials

The following are available online at: https://www.mdpi.com/article/10.3390/plants14030382/s1. Figure S1. Phylogenetic analysis of *Trichoderma asperellum* TaMFP1 and TaMFP2.
