# Supplementary figures and images for: Potential of Trichoderma asperellum as a Growth Promoter in Hydroponic Lettuce Cultivated in a Floating-Root System
Source: Plants (Basel). 2025 Jan 26;14(3):382. doi: 10.3390/plants14030382 (PMC11821087; doi:10.3390/plants14030382)

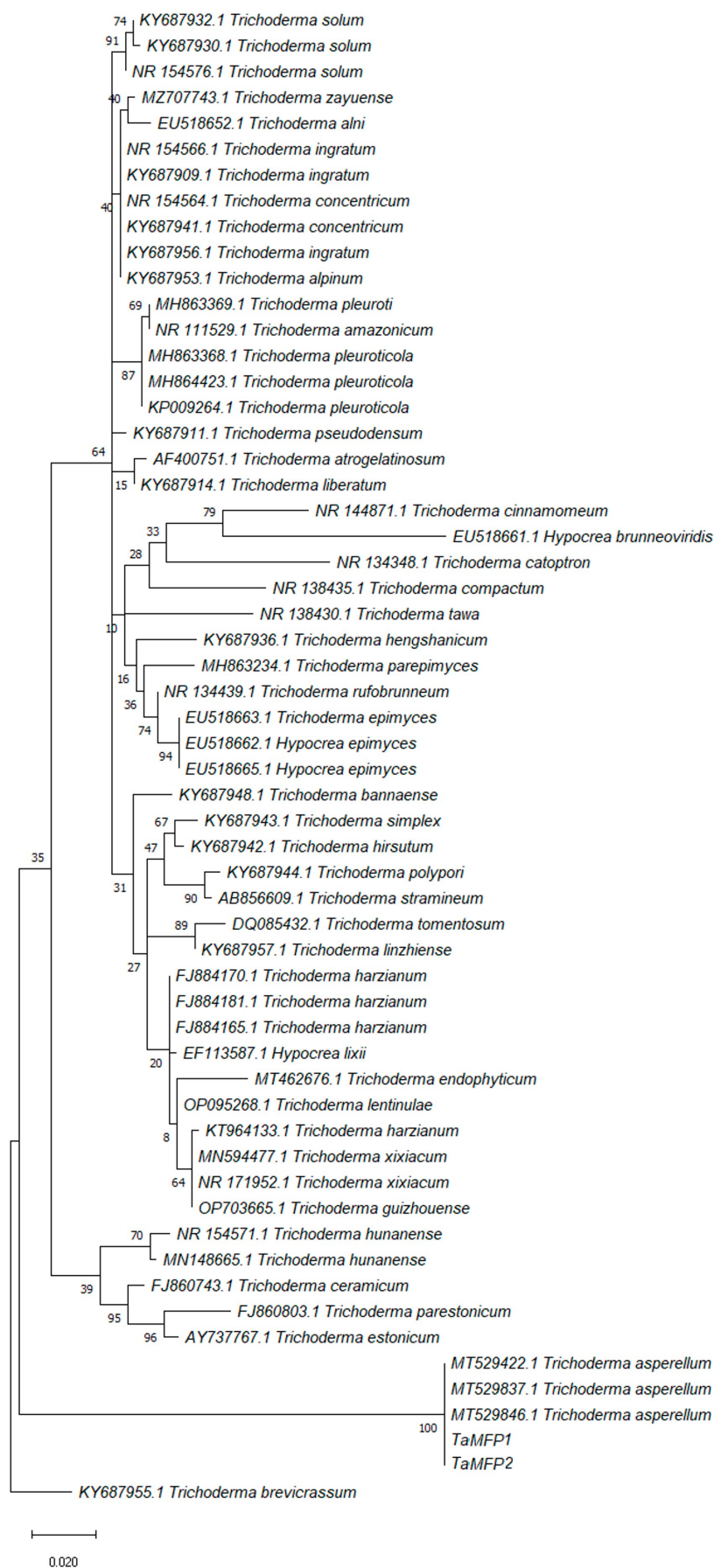

Figure S1. Phylogenetic analysis of *Trichoderma asperellum* TaMFP1 and TaMFP2.

Supplement: Supplementary file 1 [file plants-14-00382-s001.zip › plants-3375634-supplementary.pdf]
